# A hybridization of evolution strategies with iterated greedy algorithm for no-wait flow shop scheduling problems

**DOI:** 10.1038/s41598-023-47729-x

**Published:** 2024-01-29

**Authors:** Bilal Khurshid, Shahid Maqsood, Yahya Khurshid, Khawar Naeem, Qazi Salman Khalid

**Affiliations:** 1 Department of Industrial Engineering, University of Engineering and Technology, Peshawar, 25000 Pakistan; 2 Department of Industrial Engineering, Jalozai Campus, University of Engineering and Technology, Peshawar, 25000 Pakistan; 3 College of Science and Engineering, Qatar Foundation, Hamad Bin Khalifa University (HBKU), P.O. Box: 34110, Doha, Qatar

**Keywords:** Computer science, Engineering

## Abstract

This study investigates the no-wait flow shop scheduling problem and proposes a hybrid (HES-IG) algorithm that utilizes makespan as the objective function. To address the complexity of this NP-hard problem, the HES-IG algorithm combines evolution strategies (ES) and iterated greedy (IG) algorithm, as hybridizing algorithms helps different algorithms mitigate their weaknesses and leverage their respective strengths. The ES algorithm begins with a random initial solution and uses an insertion mutation to optimize the solution. Reproduction is carried out using (1 + 5)-ES, generating five offspring from one parent randomly. The selection process employs (µ + λ)-ES, allowing excellent parent solutions to survive multiple generations until a better offspring surpasses them. The IG algorithm’s straightforward search mechanism aids in further improving the solution and avoiding local minima. The destruction operator randomly removes d-jobs, which are then inserted one by one using a construction operator. The local search operator employs a single insertion approach, while the acceptance–rejection criteria are based on a constant temperature. Parameters of both ES and IG algorithms are calibrated using the Multifactor analysis of variance technique. The performance of the HES-IG algorithm is calibrated with other algorithms using the Wilcoxon signed test. The HES-IG algorithm is tested on 21 Nos. Reeves and 30 Nos. Taillard benchmark problems. The HES-IG algorithm has found 15 lower bound values for Reeves benchmark problems. Similarly, the HES-IG algorithm has found 30 lower bound values for the Taillard benchmark problems. Computational results indicate that the HES-IG algorithm outperforms other available techniques in the literature for all problem sizes.

## Introduction

The no-wait flow shop scheduling problem (NWFSSP) is a variant of the classic permutation flow shop scheduling problem (PFSSP), and it finds application in several industrial domains, including hot metal rolling, chemical processing, food production, pharmaceuticals, and plastic manufacturing, among others^1^. Permutation flow shop scheduling involves processing various jobs in the same sequence on a defined set of machines, resulting in wait times for jobs before machine allocation as well as idle times for machines. However, some crucial and practical jobs need to be processed with specific constraints. Examples of such constraints include the absence of a buffer storage facility between intermediate machines, which may cause blocking, and the need for continuous processing of a particular job, which can result in no-wait scheduling issues. In NWFSSP the consecutive operations of jobs are performed without any interruption, hence a job cannot be stored in a buffer or held on a machine^2^. Interest in NWFSSP began in the 1970s and has continued to grow due to its practical implementation in various manufacturing environments. This is particularly relevant in manufacturing processes that are dependent on specific temperatures or other conditions, and thus cannot be stored in a buffer. Other factors that support the use of NWFSSP include the ability to achieve lean production and reduce work-in-process inventory^3^. Moreover, the NWFSSP problem is classified under the NP-hard problem category, and it is considered one of the most challenging scheduling problems, even when there are only two machines involved^4^.

Numerous authors highlighted the practical applications of NWFSSP in advanced industries, including the Steel industry^5,6^, Patient and Surgery scheduling problems^7,8^. Flight scheduling^9,10^, Parallel computing^11,12^, Traffic control system^13^, Train Scheduling^14^, Bakery production^15^, Robotic cells^16,17^, and Glass manufacturing^18^. The NWFSSP concept can be illustrated by considering a steel factory where heated metal must undergo continuous processing to maintain its temperature. In this scenario, each job must also undergo continuous processing in a no-wait flow shop. Once a job starts processing on the first machine, it must proceed through all machines in the factory without any interruptions or pauses to satisfy the no-wait constraint. Any delay in the processing of a job to fulfill the no-wait constraint will result in a delay in its completion. The most frequently studied optimization criteria for NWFSSP are total completion time and makespan^19^. The makespan is the total time needed for all job operations to be completed before they exit the system^20^. Minimizing the makespan is crucial for enhancing resource efficiency and completing a batch of jobs as quickly as possible. The total completion time is the sum of all completion times, and minimizing it is beneficial for increasing the processing rate. This approach is suitable for reducing the work-in-process inventory and for quickly meeting demand requirements.

This study aims to employ the HES-IG algorithm to minimize the makespan of NWFSSP. ES is a population-based technique and it can solve scheduling problems with large search spaces, however, it can get stuck in local minima and can suffer from premature convergence. To resolve this issues the ES algorithm is combined with the IG algorithm, which can generate high-quality solutions by combining with other meta-heuristics and also avoids local minima. Minimization of makespan is the most common objective function studied by researchers since it leads to maximum utilization of machines and the manufacturing costs are also reduced. For further details on NWFSSP, the readers should follow the review papers of Hall and Sriskandarajah^5^, Allahverdi^3^, and Allahverdi et al.^2^. The paper is structured as follows: “Literature review” section contains an extensive review of relevant literature on NWFSSP, as well as the application of ES and IG algorithms in resolving other optimization problems. In “Problem statement” section, the problem statement is presented. The methodology is described in “Methodology” section, while “Results” section presents and analyzes the experimental results. Lastly, “Conclusion, future work, and limitations” section presents the conclusion, proposes potential avenues for future research and limitations.

## Literature review

NWFSSP is a combinatorial optimization problem, its complexity increases significantly as the size of the problem grows. As a result, resolving larger instances of the problem becomes increasingly difficult. Solving NWFSSP using exact methods i.e. Branch and bound method, mixed integer programming, etc. is difficult due to the complex nature of these problems. Hence Meta-heuristic algorithms i.e. Ant colony optimization (ACO), Simulated annealing (SA), Particle swarm optimization (PSO), Tabu search (TS), and Genetic algorithm (GA), among others are used to solve these complex problems as they have successfully solved other complex problems. Hall and Sriskandarajah^5^ carried out an extensive survey of various solution techniques used to solve NWFSSP, the survey paper covered all solution techniques used until 1993. Allahverdi^3^ presented another survey paper on NWFSSP and covered all solution techniques used for NWFSSP from 1993 to 2016. Hence, the readers should read the above-mentioned papers to review various solutions and techniques used for solving NWFSSP until 2016. Engin and Günaydin^21^ utilized an adaptive learning approach to minimize makespan of NWFSSP. A simple heuristic algorithm based on a GA was suggested by Silva et al.^22^ to minimize the makespan of NWFSSP. The algorithm uses permutation representation and an intensive local search utilizing insert and swap moves to find better solutions. Based on an iterated search method, Mousin et al.^23^ proposed a new approach using a sub-sequence of consecutive jobs to minimize the makespan of NWFSSP. During solution search, the combinatorics of the initial problem is first reduced and then increased. For distributed NWFSSP, Komaki and Malakooti^24^ presented a general variable neighborhood search method (GVNS). Based on crossover and mutation mechanism, Engin and Güçlü^25^ suggested an effective hybrid ant colony algorithm to minimize makespan of NWFSSP.

The GVNS algorithm, similar to the variable neighborhood search technique, involves a local search algorithm and a shaking procedure, although the shaking procedure’s intensity varies based on the solution’s advancement. Additionally, by adopting time-saving techniques, the GVNS concentrates on potential solutions that show promise. Shao et al.^26^ proposed a hybrid metaheuristic approach based on the probabilistic teaching–learning mechanism (PTLM) to solve NWFSSP, to minimize the makespan. The PTLM comprises four primary components: previewing before the class, the teaching phase, the learning phase, and the review after the class. The previewing stage employs NEH heuristic and opposition-based learning, while the teaching phase utilizes the Gaussian distribution to guide the search toward promising regions. The learning phase involves the use of crossover, and for reviewing after the class, the authors applied simulated annealing with an improved speed-up random insert local search to enhance the algorithm’s local search capabilities. A hybrid meta-heuristic based on improved ACO and SA is presented by Riahi and Kazemi^27^ for NWFSSP. The approach adopted in this study employs ACO to create solutions and update the pheromone trail to obtain the optimal solution. Subsequently, SA is used to refine the solution further and identify the most favorable neighborhood solution. Lin et al.^28^ suggested a cloud theory-based IG algorithm to minimize makespan and the weighted sum of total tardiness of NWFSSP. Using dominance relations, Allahverdi et al.^2^ introduced an AA algorithm, which is a hybridization of SA and the insertion algorithm. The algorithm’s primary objective is to minimize both the makespan and the total tardiness. Zhu et al.^29^ proposed a novel quantum-inspired cuckoo co-search algorithm to minimize the makespan of the NWFSSP. The algorithm comprises three phases, which are as follows: (1) a Quantum representation of the solution, (2) A quantum-inspired cuckoo search-differential evolution search, and (3) a Local neighborhood search algorithm. Furthermore, the algorithm’s convergence property was analyzed theoretically.

Zhao et al.^30^ proposed a hybrid algorithm called HBV, which combines biogeography with variable neighborhood search (VNS) to minimize the makespan of NWFSSP. The initial solution for the HBV algorithm is created by employing the NEH heuristic and the nearest neighborhood mechanism. To accelerate the convergence of the algorithm, a hybrid migration operator is combined with the path relink technique. To enhance the exploitation ability of the algorithm, the mutation operator is integrated with an iterated greedy algorithm. A variable neighborhood strategy based on insert neighborhood structure and block neighborhood structure is adopted to locate the best solution in the vicinity of the current solution. Tasgetiren et al.^31^ aimed to minimize the total flow time and total energy consumption by using a mixed integer programming model to identify Pareto optimal sets, which were then tested on Taillard instances. To obtain a non-dominated set of solutions for large instances, the researchers developed three heuristics: a Discrete Artificial Bee Colony, an Energy-efficient genetic algorithm, and another variant of energy-efficient genetic algorithm. Of the three algorithms, the Discrete Artificial Bee Colony algorithm outperformed the other two in terms of finding better solutions. Zhao et al.^32^ proposed a factorial-based particle swarm optimization (PSO) method for solving the NWFSSP by incorporating a population adaptation mechanism. The NEH heuristic and nearest neighborhood mechanism are used to generate the initial solution. A variable neighborhood structure based on swap and insert neighborhood is employed to find the best solution around the current solution. To prevent being stuck in local optima and maintain population diversity, the population adaptation mechanism is used. Shao et al.^33^ addressed a multi-objective distributed NWFFSP with sequence-dependent setup time, using makespan and total weight tardiness as performance criteria. To achieve this, a Pareto-based estimation of the distribution algorithm was employed, along with the construction of three probabilistic models. These models included the probability of jobs in an empty factory, two jobs in the same factory, and adjacent jobs. The algorithm was extended to a distributed environment to generate initial individuals. Additionally, a sampling method with a referenced template was introduced to generate offspring individuals, and several multi-objective neighborhood search methods were developed to optimize solution quality.

To minimize makespan in the food industry with release date constraints, Pourhejazy et al.^34^ proposed two algorithms i.e. beam search method and a local search based-beam search method, and tested them on benchmark problems of Taillard, Vallada, and Reeves. The improved beam search algorithm found better solutions as it avoided early convergence and local optimality by dismissing non-prominent solutions. Zhao et al.^35^ introduced a Jigsaw puzzle heuristic (JPA) for minimizing the makespan of NWFSSP. The JPA uses a waiting time matrix to evaluate the distance between two jobs. Subsequently, a matching matrix is generated from the waiting time matrix. The final solution is then created based on the matching matrix. Wu and Che^36^ proposed an adaptive multi-objective variable neighborhood search for simultaneous minimization of makespan and total energy consumption in NWFSSP. Two heuristics are designed initially to minimize total energy consumption without increasing makespan. The descent phase of the variable neighborhood search method integrates insertion and swap moves. To enhance the algorithm’s performance, a novel problem-specific shake procedure is employed. Accelerated techniques are used to increase the algorithm’s speed. According to Zhao et al.^37^ research, a two-stage cooperative evolutionary algorithm (TS-CEA) was proposed as a solution to energy-efficient scheduling of the NWFSSP, aiming to minimize both makespan and total energy consumption. To create an initial solution, two constructive heuristics were designed by analyzing the problem’s properties. TS-CEA uses an iterative local search strategy to explore potential extreme solutions in the first stage, along with a hybrid neighborhood structure that enhances the quality of the solution. In the second stage, a mutation strategy based on critical path knowledge was proposed to extend the extreme solutions to the Pareto front.

Yüksel et al.^1^ introduced a mixed-integer linear programming model (MILP) and a constraint programming model for minimizing total energy consumption and total tardiness in bi-objective NWFSSP. As finding the solution for total tardiness is an NP-hard problem, the authors proposed multi-objective algorithms such as a genetic algorithm, discrete artificial bee colony algorithm, and GA integrated with local search to address the bi-objective NWFSSP. Xuan et al.^38^ proposed a novel genetic SA algorithm for minimizing total flow time in unrelated parallel machines. The NEH heuristic is used for generating the initial solution. A two-dimensional matrix encoding is used for solution design, followed by an insertion translation approach for decoding. To avoid premature convergence and enhance exploration ability, an adaptive adjustment strategy is applied in the crossover and mutation operators. To improve the performance of the GA, SA with five search methods is used. To minimize the completion time of the assembly process in distributed assembly NWFSSP, Zhao et al.^39^ proposed a backtracking search algorithm in which the initial solution was generated from three heuristics. The suggested approach of block-shifting ensures that the best possible subsequence of a potential solution is preserved during the mutation process. Moreover, the similarity among candidate solutions is employed as a feedback indicator to regulate the application of block-shifting.

Miyata and Nagano^40^ addressed the issue of minimizing makespan in the distributed NWFSSP with sequence-dependent setup times and maintenance operations. A mixed-integer linear programming (MILP) formulation is employed to formally represent the problem, and heuristic methods are proposed to incorporate maintenance operations into job scheduling. For Distributed NWFSSP, Shao et al.^41^ proposed a MILP to minimize the makespan. This study introduces a machine selection technique that follows the “first earliest available machine” rule. In addition, three factory assignment rules, which use the NEH (Nawaz-Enscore-Ham) heuristic, are proposed to allocate jobs to factories in a greedy manner. Furthermore, a set of 14 dispatch rules, based on simple sorting and decomposition, are developed to establish a priority sequence for jobs. By combining these dispatch rules and factory assignment rules, several constructive heuristics are derived. Keskin and Engin^42^ tackled the issue of scheduling in the NWFSSP with setup times, focusing on two performance metrics: total flow time (ΣCj) and makespan. To address this problem, a Hybrid Genetic Local and Global Search Algorithm was introduced. This hybrid genetic algorithm was formulated by combining an insert-search approach with a self-repair algorithm featuring a self-repair function. Considering a fuzzy environment in NWFSSP, Başar and Engin^43^ used an improved Scatter search approach to minimize the makespan with setup times. Initially, the effectiveness of the proposed algorithm is evaluated on benchmark problems in the literature. Following this, the proposed improvement scatter search algorithm for fuzzy due dates is applied to solve a benchmark NWFSSP that includes setup times. Azerine et al.^44^ investigated the two-machine NWFSSP with two competing agents, aiming to minimize the overall completion time of one agent while adhering to an upper bound on the makespan of the second agent. Polynomial-time algorithms were demonstrated for certain restricted scenarios. Additionally, a mathematical programming model and a branch and bound approach were suggested as solving methods for smaller problems. For larger instances, a tabu search meta-heuristic algorithm was developed.

A mixed integer linear programming model is proposed by Zeng et al.^45^ to minimize the total energy consumption and makespan of PFSSP with no-wait constraints and sequence dependent setup times. The initial solution is generated using NEH heuristic and then the solution is optimized using Improved Non-dominated Sorting Genetic Algorithm. Additionally, this author introduced two heuristics for adjusting speed tailored to the specific problem, aiming to improve the quality of the non-dominated solutions acquired. Karacan et al.^46^ thoroughly examined the NWFSSP with the primary aim of optimizing both earliness (E) and tardiness (TT) objectives. To tackle this intricate challenge, he introduced a novel perspective on enhancing the parallel simulated annealing algorithm. This inventive approach is specifically designed to alleviate the runtime limitations commonly linked with traditional simulated annealing while simultaneously reinforcing its resilience. To address multi-objective challenge of energy-efficient distributed assembly NWFSSP, Zhao et al.^47^ introduces a novel approach called reinforcement learning-driven brain storm optimization to minimize the makespan and total energy consumption (TEC), and achieving balanced resource allocation. To optimize the objective of maximum assembly completion time, the study incorporates four critical operations: critical factory insertion, critical factory swapping, critical factory insertion into other factories, and critical factory swapping with other factories. Utilizing the simple structure of Iterated local search (ILS) algorithm, Avci^48^ minimized the makespan of distributed NWFSSP by combining two variable neighborhood descend based procedures with the ILS algorithm. Furthermore, the perturbation intensity is dynamically tailored to match the characteristics of the search space’s structure.

ES adopts a hill-climbing procedure and is developed to solve numerical optimization problems. Recently ES has been applied to discrete optimization problems. Evolutionary algorithms can be divided into subclasses, namely ES and GA. While both techniques use the principle of selecting the fittest individual and maintaining populations of feasible solutions, there are significant differences between them. One key distinction is in the way individuals are represented: GA uses binary vectors, whereas ES uses floating-point vectors. Another difference is the selection procedure, with GA using a random selection process while ES relies on a deterministic approach. Additionally, the relative order of procedures is different, with selection preceding recombination in ES, and recombination preceding selection in GA. Overall, while both techniques share some similarities, they also have notable differences. ES has proven to be effective in addressing a wide range of optimization problems i.e. Distributed blocking FSSP^49^, Image segmentation^50^, Permutation FSSP^51,52^, Blocking FSSP^53^, Multiple traveling salesman problem^54^, Robust Permutation FSSP^55^, Team orienting problem^56^, Open vehicle routing^57^, Group scheduling problem^58^, Assembly line balancing^59^. Keeping in view the successful results obtained by using ES for various optimization problems, in this research work ES is designed and applied to NWFSSP to minimize the makespan. The ES algorithm can be computationally expensive, suffer from premature convergence, and have suboptimal solution quality for multi-objective solutions. These issues can be addressed by combining the ES algorithm with another local search method to enhance its performance.

The optimal meta-heuristic for PFSSP is the Iterated Greedy (IG) algorithm, as proposed by Ruiz and Stützle^60^ due to its ability to yield superior solutions using a straightforward search method. The IG algorithm is composed of two phases: a destruction phase, during which elements are eliminated from the solution, and a construction phase, during which a greedy constructive heuristic is used to reintroduce the eliminated elements. Further improvement to the solution is made in the reconstruction phase through local search. An enhanced version of the IG algorithm, with a Tabu reconstruction strategy, was introduced by Ding et al.^61^ for NWFSSP. The researchers applied the improved algorithm to Taillard instances and identified 43 new upper bounds. The efficacy of the algorithm is primarily attributed to the reconstruction strategy, which enhances the exploration ability and steers the search towards more advantageous regions by avoiding local minima. To further refine the solution, the neighborhood search method incorporates swap, inset, and double insert moves. The IG algorithm is a highly adaptable algorithm that can be customized to suit various scheduling problems and integrate a range of local search methods. By repeatedly combining solutions from different heuristics or perturbations, it can rapidly generate top-quality solutions. Moreover, the algorithm can quickly converge to a near-optimal solution, particularly when searching through a vast solution space. IG algorithm has a simple implementation architecture, often performs well in resolving FSSPs, and offers a lot of potential for use in resolving industrial issues^62^. IG algorithm has been successfully applied to solve different scheduling problems i.e. Permutation flow shop scheduling problems, Distributed flow shop scheduling problems^63–65^, No-wait flow shop scheduling problems^66^, Blocking flow shop scheduling problems^67,68^, No idle flow shop scheduling problems^69^, Hybrid Flow shop scheduling problems^70^. Hybrid IG algorithms are also used to solve different Flow Shop Scheduling problems (FSSP) in which the IG algorithm is combined with other heuristics i.e. GA^71^, VNS^72^, Artificial Bee Colony^73^, Differential Evolution^74^, Teaching learning based optimization (TLBO)^75^, and Water wave optimization algorithm^76^. For further details of the IG algorithm, the reviewers should read the paper of Zhao et al.^62^.

The IG algorithm is a computationally intensive method that involves executing multiple local searches and combining the solutions. It may encounter difficulties in overcoming local minima, particularly when the initial solution is suboptimal or the search space is not adequately explored. The effectiveness of the algorithm is highly dependent on the quality of the local search algorithms used in each iteration. If the local search algorithm is not proficient, the overall performance of the IG algorithm may be subpar. By combining different algorithms, hybridization can improve the overall performance of the resulting hybrid algorithm in terms of solution quality, convergence speed, robustness, and scalability. Hybridization can also help to tackle complex real-world problems that cannot be solved by a single algorithm alone. The combination of complementary techniques from different algorithms can often lead to superior results compared to any single algorithm. To utilize the strength and overcome the weaknesses of the IG algorithm, it is combined with the ES algorithm, the resultant HES-IG algorithm helps in avoiding local minima. The IG algorithm starts with the improved solution provided by the ES algorithm, hence the IG algorithm starts with an already improved solution and it takes less time to provide a very feasible solution. In Table 1, various solution techniques used for NWFSSP are summarized.

**Table 1:** Solution techniques used to minimize the makespan of NWFSSP.

Author	Problem	Technique	Makespan	Instances	Coding language
Ding et al.^61^	*F*_*M*_* |No wait|C*_*max*_	Tabu mechanism improved IG algorithm	✓	Reeves, Taillard	C++
Silva et al.^22^	*F*_*M*_* |No wait|C*_*max*_	Simple heuristic algorithm	✓	Reeves	C
Mousin et al.^23^	*F*_*M*_* |No wait|C*_*max*_	IG algorithm	✓	Taillard	C++
Komaki and Malakooti^24^	*F*_*M*_* |No wait, dist|C*_*max*_	General VNS	✓	Naderi (Taillard)	Matlab
Shao et al.^26^	*F*_*M*_* |No wait|C*_*max*_	Probabilistic TLBO	✓	Reeves, Taillard, Vallada	Anova
Riahi and Kazemi^27^	*F*_*M*_* |No wait| TT, C*_*max*_	ACO and SA	✓	Reeves, Taillard	Delphi
Lin et al.^28^	*F*_*M*_* |No wait| ∑wT, C*_*max*_	Cloud theory-based IG algorithm	✓	Carlier, Reeves, Taillard	C++
Allahverdi et al.^2^	*F*_*M*_* |No wait| TT, C*_*max*_	SA, Insertion algorithm	✓	Self-generated	Anova
Zhu et al.^29^	*F*_*M*_* |No wait |C*_*max*_	Novel quantum-inspired cuckoo co-search algorithm	✓	Reeves	VC++
Zhao et al.^30^	*F*_*M*_* |No wait |C*_*max*_	Hybrid biogeography, VNS	✓	Taillard, Vallada	Matlab
Tasgetiren et al.^31^	*F*_*M*_* |No wait |, TF, TEC*	Discrete ABC, variants of Energy efficient GA		Taillard	C++
Zhao et al.^32^	*F*_*M*_* |No wait |C*_*max*_	Factorial-based PSO	✓	Reeves, Taillard	Anova
Zhao et al.^35^	*F*_*M*_* |No wait |C*_*max*_	Jigsaw puzzle heuristic	✓	Taillard, Vallada	Matlab
Pourhejazy et al.^34^	*F*_*M*_* |No wait, r*_*j*_* |C*_*max*_	Beam search method	✓	Taillard, Vallada, Reeves	C++
Yüksel et al.^1^	*F*_*M*_* |No wait| TT, TEC*	MILP model		Taillard	C++
Wu and Che^36^	*F*_*M*_* |No wait| C*_*max*_*, TEC*	Multi-objective neighborhood search	✓	Self-generated	C++
Zhao et al.^37^	*F*_*M*_* |No wait |C*_*max,*_* TEC*	Two stage cooperative evolutionary algorithm	✓	Taillard	Matlab
Keskin and Engin^42^	*F*_*M*_* |No wait| C*_*max*_*,* ΣCj	Hybrid Genetic Local and Global Search Algorithm	✓	Engin and Günaydın	–
Xuan et al.^38^	*F*_*M*_* |No wait |, TF*	Novel SA algorithm		Self-generated	Matlab
Başar and Engin^43^	*F*_*M*_* |No wait| C*_*max*_	Scatter search	✓	Self-generated	C++
Zeng et al.^45^	*F*_*M*_* |No wait |C*_*max,*_* TEC*	Improved Non-dominated Sorting Genetic Algorithm	✓	Self-generated	PlatEMO
Karacan et al.^46^	*F*_*M*_* |No wait | E, TT*	SA algorithm		Carlier	C#
Zhao et al.^47^	*F*_*M*_* |No wait | C*_*max,*_* TEC*	Reinforcement learning-driven brain storm optimisation idea	✓	Self-generated	–
Avci^48^	*F*_*M*_* |Dist, No wait | E, TT*	Iterated local search algorithm	✓		C++

## Problem statement

The key difference between PFSSP and NWFSSP is that in PFSSP, a job can be retained on a machine or within two machines while in NWFSSP job cannot be retained on any machine or within machines. The study aims to minimize the makespan for NWFSSP and find an optimal sequence so that all jobs are processed. Due to technological constraints, there are cases where jobs cannot be retained on a machine or within machines. Hence, when a job is started, it remains uninterrupted till the last operation on the last machine is completed.

In NWFSSP, n-jobs are processed in the same sequence on m-machines in the same order. Each job j (j = 1, 2,…,n) has a predefined processing time p_i,j_ on machine I (i = 1, 2, …, m). The goal of NWFSSP is to determine an optimal order for completing all jobs on all machines termed as makespan and denoted as C_max_ or C_m,n_, such that there is no waiting time for any job, and all jobs follow the same sequence. This problem makes several assumptions, including that each machine can handle only one job at a time, each job can only be processed by one machine at a time, all jobs follow the same processing sequence, and every machine processes jobs in the same order. Additionally, no waiting time is permitted on or between machines, and a job cannot be interrupted once it has begun until its final operation is complete. Processing times are known in advance, and any job can start at time zero, with preemption not being permitted.

The no-wait feature of this problem guarantees that the time it takes to complete one job and start the next job is solely influenced by the processing times of these two jobs, without regard to the other jobs in the sequence. Consequently, it is possible to establish a completion time separation for every pair of jobs. To determine the completion time distance from job i to job j, it can be calculated as follows^61^:

1 $${D}_{i,j}=\genfrac{}{}{0pt}{}{max}{k=1,\dots ,m}\left\{\sum_{h=k}^{m}\left({p}_{h,j}-{p}_{h,i}\right)+{p}_{k,i}\right\}.$$

The completion time distances can be calculated in advance. A schedule for the NWFSSP can be represented as a job permutation π = [π(1), π(2),…, π(n)], where π(k) ∈ {1,2,…, n} and π(k) ≠ π(kʹ), ∀ k ≠ kʹ. The makespan of a feasible schedule can be calculated using Eq. (2).

2 $${C}_{max}\left(\pi \right)={C }_{m,n}\left(\pi \right)=\sum_{j=2}^{n}{D}_{\left[j-1\right],\left[j\right]}+ \sum_{k=1}^{m}{p}_{\pi \left(1\right),k}.$$

*D*_[j−1],[j]_ represents the completion time distance between (j − 1)th and *j*th jobs in schedule π, i.e. *D*_π [j−1], π [j]_. A dummy job π(0) having zero processing time is introduced in the permutation π to simplify the makespan expression. Hence the schedule can be updated as π = [π(0), π(2),…,π(n)]. The makespan of a permutation π can be calculated using Eq. (3).

3 $${C}_{max}\left(\pi \right)={C }_{m,n}\left({\pi }{\prime}\right)=\sum_{i=1}^{n}{D}_{\left[j-1\right],\left[j\right]},$$

where

4 $${D }_{\left[0\right],\left[1\right]}=\sum_{k=1}^{m}{p}_{\pi \left(1\right),k}.$$

If ∏ denotes the set of all possible permutation schedules. Then the objective is to find a permutation schedule π^*^ ∈ ∏ such that:

5 $${C}_{max}\left({\pi }^{*}\right)={\genfrac{}{}{0pt}{}{min}{\uppi \in \prod } C}_{max}\left(\pi \right).$$

## Methodology

### Evolution strategies

ES is a subclass of the evolutionary algorithm, which mimics the principles of natural evolution for solving parameter optimization problems. ES was developed in the 1960s by Rechenberg^77^ and Schwefel^78^. ES was previously believed to be evolutionary algorithms that exclusively utilized mutation as a recombination operator and were represented by floating-point numbers. However, their application has expanded to a range of optimization problems that involve constantly changing parameters, including discrete optimization problems. The first ES algorithms relied on a single population and a single genetic operator, namely mutation, in the evolution process. Nevertheless, the idea of representing the individual as a pair of float-valued vectors was fascinating. In the “two-membered ES”, an offspring competes with its parent during the competition stage, resulting in two individuals in the population.

In multi-membered ES, the size of the population is greater than one. In addition, the mating probability for the individuals is the same. A recombination operator is also used to generate offspring from two parents. Both the multi-membered and the two-membered have one thing in common i.e. they produce a single offspring. A convenient notation of the ES is as under:

- Two-membered evolution strategy (1 + 1)-ES.
- Multi-membered evolution strategy (µ + 1)-ES.

where µ represents the population size.

**Figure 1:**
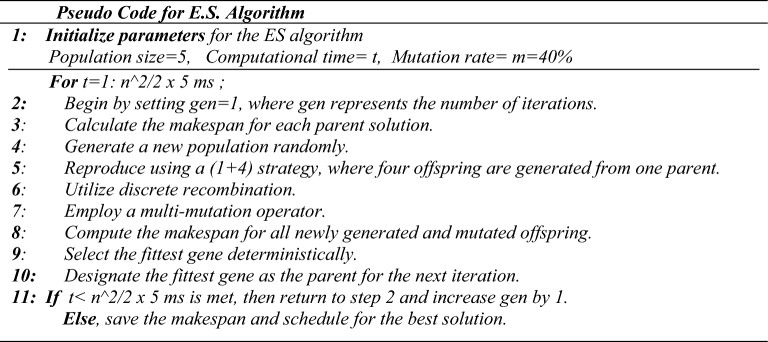
Pseudo code for the ES algorithm.

The multi-membered ES evolved further to mature as (µ + λ)-ES and (µ, λ)-ES. The (µ + λ)-ES is an extension of the multi-membered-ES (µ + 1)-ES, where offspring (λ) are produced from individuals (µ). A selection process then reduces the population of (µ + λ) individuals to µ individuals. While λ offspring are produced from µ individuals in (µ, λ)-ES, and the selection process produces λ offspring from a new population of λ individuals. The ES algorithm involves generating a population of offspring that undergoes mutation and evaluation of their objective function during each iteration. The better-performing offspring are then recombined to create a new population for the subsequent iteration. This procedure is continued until the termination criteria are satisfied. The ES algorithm distinguishes itself from other evolutionary algorithms in its utilization of mutation and recombination operators, as well as its unique representation of the population. Figure 1 shows the pseudo-code for the ES algorithm.

#### Initial solution

To implement parent selection in the ES algorithm, a uniform distribution is utilized to randomly choose parents from the population. This approach guarantees that each individual in the population has an equal likelihood of being selected as a parent. Heuristics can also be used to create the initial solution, however, it makes the algorithm complex and increases the computational time.

#### Mutation operator

**Figure 2:**
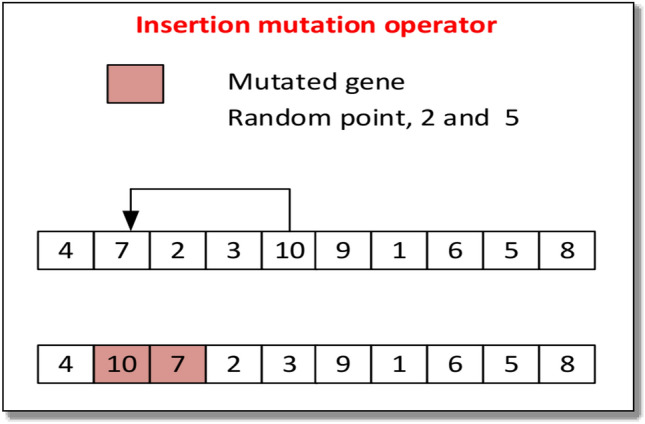
Procedure for insertion mutation.

The mutation operator in the ES algorithm serves to introduce genetic diversity into the parent population, making it a vital source of genetic variation. The mutation is mandatory to ensure that the new offspring is different from its parent. The diversity is controlled using the mutation rate. Different types of mutation operators are available i.e. Insertion mutation, scrambled mutation, swap mutation, and inversion mutation, among others. Since insertion mutation performs best for Flow shop scheduling problems (FSSP). Hence we are using insertion mutation in this research work. Unlike other mutation operators, insertion mutation does not disrupt the existing structure of the algorithm, which can help to maintain the integrity of the genetic information^79^. The procedure of the insertion mutation is shown in Fig. 2.

#### Reproduction operator

**Figure 3:**
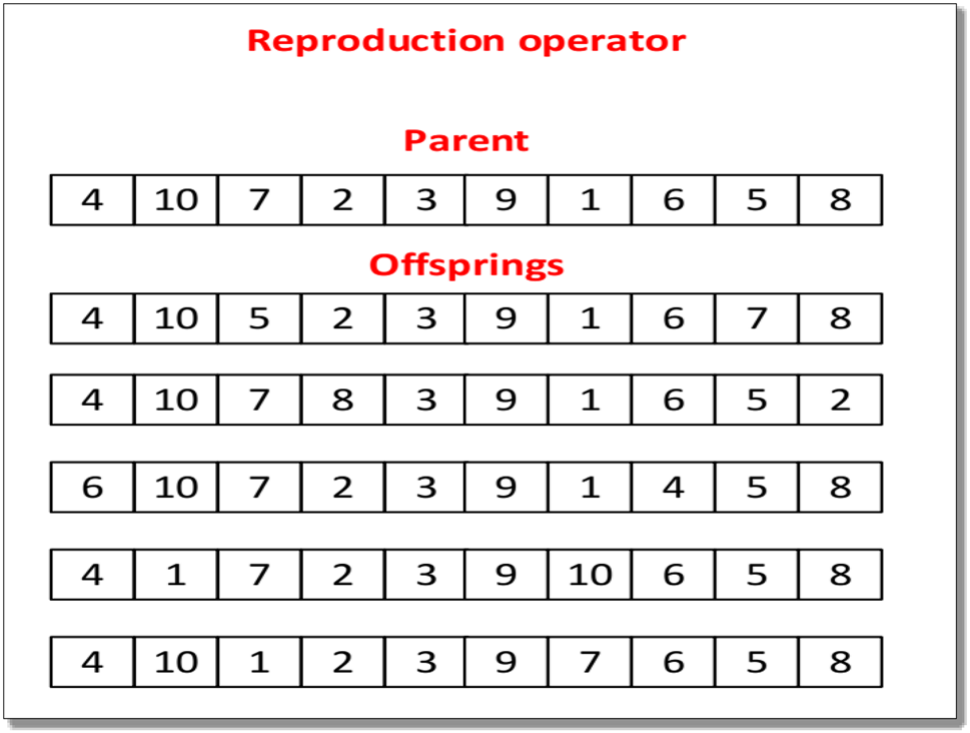
Procedure for reproduction operator.

In reproduction, λ offspring are generated from µ individuals. Different types of reproduction operators are available i.e. 1, 4, 5, 9, and 16^80^. It should be noted that varying the value of λ in the algorithm affects the computational time and the number of offspring generated from a single parent. Specifically, setting λ to 1 results in the minimal computational time since only one offspring is created. However, increasing λ to 4, 5, 9, or 16 will generate four, five, nine, or sixteen offspring from a single parent, respectively. Notably, when λ is set to 16, the algorithm explores the maximum solution space, but this comes at the cost of increased computational time. To save computational time and find a better solution in the search space, λ = 5 is used in this research paper. Hence from one parent five offspring are randomly generated. The procedure of the reproduction operator is shown in Fig. 3.

#### Survivor selection operator

During survivor selection, the best individuals are deterministically selected from the descendent, and their fitness value is calculated. There are two types of survivor selection i.e. (µ + λ) and (µ, λ). The (µ + λ)-selection method involves selecting the top offspring (µ) from the combined pool of parents and offspring. This approach is considered elitist since a parent can persist for several generations if none of its offspring can surpass its fitness. However, the (µ, λ) strategy performs better in probabilistic environments, while the (µ + λ) approach is more effective for combinatorial optimization problems. In this research work, we have used (µ + λ), as (µ, λ) descendent can produce the worst results.

#### Termination criteria

In the ES algorithm, various termination criteria can be employed such as computational time, number of generations, or lack of improvement for a certain number of generations. In this research, we have chosen to use computational time as the termination criteria, with a time limit of n^2^/2 × 10 ms for each instance.

#### Hybrid evolution strategies— iterated greedy algorithm (HES-IG)

The purpose of hybridizing algorithms is to combine two or more different algorithms or techniques to leverage their respective strengths and mitigate their weaknesses^61^. This approach aims to create a new algorithm that performs better than its individual components in solving a specific problem or class of problems. Hybridization maintains balance between exploration and exploitation; it increases the robustness and improves the overall performance of the algorithm. Hybrid algorithms provide better results interms of speed, convergence and accuracy. However, hybridization can increase the complexity of the algorithm as managing and tuning hybrid algorithm can increase the computational resources. Other key factors which needs special attention in hybridization are (1) algorithms integration, (2) Interpretation, (3) Overfitting and (4) Increased development time. The most crucial non-trivial effort, however, is choosing the appropriate combination of algorithms and determining how they should interact. Doing so can assist to mitigate the disadvantages of hybridization.

The ES algorithm is known for its simplicity, flexibility, scalability, robustness, and effectiveness in global search^81,82^. However, it also has certain limitations such as getting trapped in local minima, slow convergence, and sensitivity to genetic operators^83^. To overcome these limitations, this paper proposes a hybrid algorithm that combines the strengths of both ES and IG algorithms. The IG algorithm is known for its simplicity and ability to find near-optimal solutions quickly and is well-suited for problems that have a preference for greedy solutions^84^. However, IG algorithm is sensitive to the initial solution. The IG algorithm exhibits clear constraints when tackling large-scale problems. Firstly, the algorithm employs a “single solution” search approach alongside a greedy insertion reconstruction technique, potentially leading to limited solution variety when compared to population-based search methods. Secondly, after completing the reconstruction phase, it typically employs a neighborhood search method, which tends to focus on exploring local minima regions. These observations suggest that enhancing the IG algorithm’s exploitation capabilities could prove advantageous^61^. The proposed hybrid HES-IG algorithm addresses these issues by first utilizing the ES algorithm to solve the problem and then incorporating the IG algorithm to further minimize the makespan more effectively and it successfully solves problems of all sizes.

Key advantages of Hybridizing ES algorithm with IG algorithm are as follows:

- *Diverse exploration and exploitation* ES is known for its ability to explore a wide solution space through the use of mutation and reproduction operators. IG, on the other hand, excels at exploiting local optima. By combining these two approaches, the hybrid algorithm can effectively balance exploration and exploitation, which is essential for finding high-quality solutions in complex optimization problems.
- *Global and local search integration* ES provides a global search capability, while IG focuses on local search and refinement. Combining these approaches allows the hybrid algorithm to first explore the global search space with ES and then fine-tune promising solutions using IG, resulting in more efficient convergence to optimal or near-optimal solutions.
- *Improved convergence speed* The hybridization can lead to faster convergence compared to using either ES or IG alone. ES can quickly generate diverse candidate solutions, and IG can refine them efficiently.
- *Parallelization* ES and IG are often amenable to parallelization. By integrating these algorithms, the hybrid approach can effectively utilize parallel and distributed computing environments, further speeding up the optimization process.
- *Reduced risk of premature convergence* ES helps mitigate the risk of premature convergence by maintaining a diverse set of candidate solutions. This diversity can prevent the algorithm from getting stuck in suboptimal regions of the search space.
- *Effective handling of large-scale problems* For large-scale optimization problems, the hybrid approach can be particularly advantageous.

**Figure 4:**
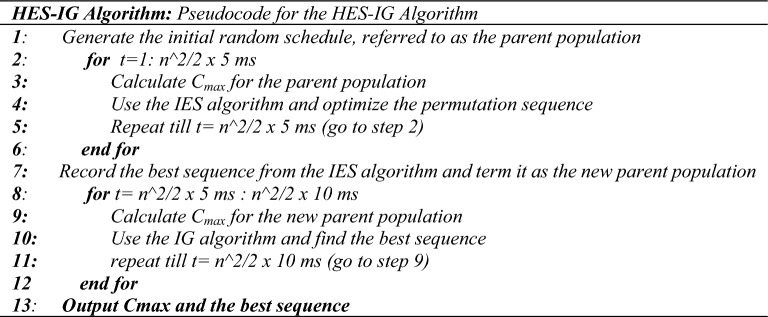
Pseudo code for the HES-IG algorithm.

The pseudo-code for the HES-IG algorithm is presented in Fig. 4.

### Iterated greedy algorithm

In 2007, Ruiz and Stützle^60^ devised an IG algorithm to solve PFSSP, which is closely related to the Iterated Local search algorithm (ILS) of Stützle^85^. Over the past 15 years, IG algorithms are used to solve different FSSPs. IG algorithm is a simple heuristic and is a single-solution-based method that starts with an initial solution and concentrates on finding additional alternatives to enhance it. IG iterates analogously across construction heuristics as opposed to a local search, as ILS does. IG algorithm iterates over greedy constructive heuristics using the two primary phases of destruction and construction to create a sequence of solutions. The other secondary phases of the IG algorithm are local search and acceptance-rejection criteria. The four phases of the IG algorithm are repeated till the termination criteria are met.

**Figure 5:**
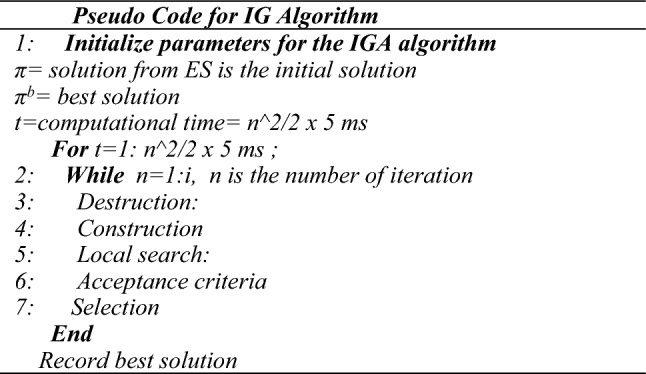
Pseudo code for the IG algorithm.

**Figure 6:**
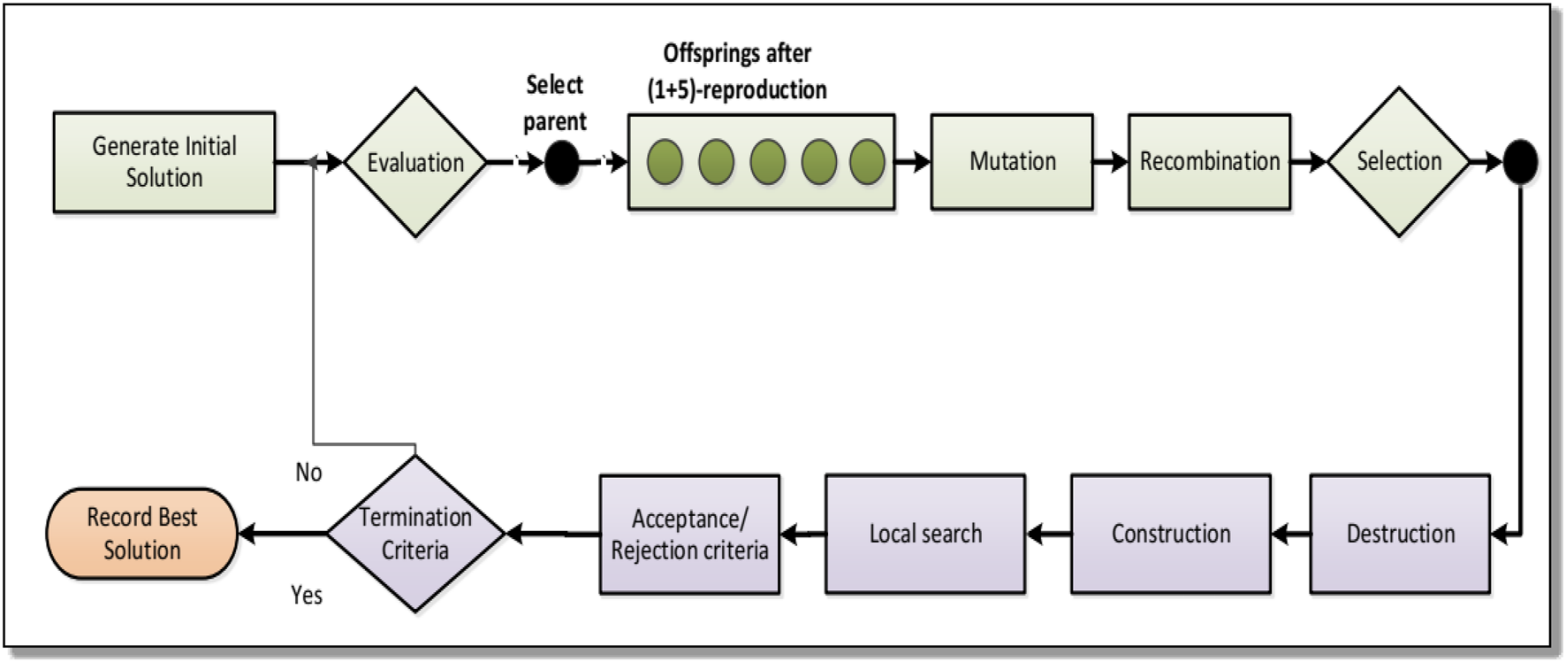
Flow chart of HES-IG Algorithm.

The key advantages of the IG algorithm are: easy to design and implement, has a flexible framework that can be combined with other algorithms, is very suitable for solving FSSP, has few parameters it can save calibration time^62^. The IG algorithm involves repeatedly applies a greedy strategy to select the locally optimal solution at each step. IG algorithm can be considered a type of iterative local search method, which repeatedly examines solutions within specific areas and utilizes perturbation techniques to avoid getting stuck in local optima. However, there are several limitations to this approach such as being Sensitive to the initial solution, being computationally expensive for large-size problems, using a single solution-based search strategy, and getting trapped in local optima^62^. However, these limitations can be addressed by combining them with other meta-heuristics such as GA, ES, SA, etc.^70^. The pseudo-code for the IG algorithm is presented in Fig. 5, while the flow chart for the IG algorithm is shown in Fig. 6 respectively.

#### Destruction

Destruction is applied to a permutation P of *n*-jobs, and it involves randomly selecting *d*-jobs without repetition, and removing them from the permutation in the order they were chosen. Destruction of sequence results in two subsequences: a partial sequence called *P*_*D*_, which consists of the remaining *n*-jobs, after the removal of *d*-jobs. The value of *d* is calculated using *d* = (n/10), for a 20-jobs case the value of d is 2, in the case of a decimal value, the value of d is rounded to the next integer value. The second partial sequence is *P*_*R*_, having a sequence of *d*-jobs. These *d*-jobs in *P*_*R*_ must be reinserted into *P*_*D*_ to create a complete candidate solution, in the order, they were removed.

#### Construction

In the construction phase also termed greedy reinsertion, the algorithm applies to step 3 of the NEH heuristic repeatedly until a complete sequence of all *n*-jobs is obtained. The process begins by using a subsequence, *P*_*D*_, and then performs *d* steps in which jobs from *P*_*R*_ are inserted into *P*_*D*_. This is done by starting with *P*_*D*_, and then inserting the first job of *P*_*R*_ (*P*_*R*_(1)) into all possible *n* + 1 positions within *P*_*D*_. The position which results in the lowest C_max_ value is then selected as the best fit for *P*_*R*_(1). This process is repeated until *P*_*R*_ is empty.

#### Local search

The local search phase is a key component in most IGAs and is vital to achieving satisfactory solutions. Different type of local search methods can be used as mentioned below:

i. Single Insertion (remove job at *i*th and place it at *j*th position).
ii. Swap (swap two neighboring jobs *i* and *i* + 1).
iii. Interchange (Interchange jobs at *i*th position with *j*th position).
iv. Multiple Insertion (remove multiple jobs and reinsert them).

Swap neighborhood methods is very fast however the solution quality is often inferior. Stützle^85^ showed that insertion neighborhood method is better as compared to Interchange method. Multiple insertion method is relatively complex as compared to single insertion method. In the parameter optimization of the IGA algorithm, Insertion method has shown better results as compared to other local search methods. In this study, we employ the Single Insertion (L1) method as our local search operator. This method improves an existing solution by removing a job and inserting it into a new position, which can be chosen as the optimal position or a random one. L1 is a widely used operator in IG algorithms and is often incorporated as a component in mixed local search operators.

#### Acceptance rejection criteria

During each iteration of the IGA, a new solution (πʹ) is generated through the destruction, construction, and optional local search phases. This new solution is compared to the previously accepted solution (π), known as the seed solution for the current iteration. Since the destruction and construction phases do not guarantee that the new solution (πʹ) will be better than the previous solution (π), an acceptance criterion is used to determine which solution will be accepted and used as the seed solution for the next iteration. The acceptance-rejection criteria in this paper are based on Stützle^85^ having a constant temperature of T adopted from the SA algorithm. The constant temperature is based on the instance number and it can be calculated using Eq. (6).

6 $$Temperature=T\times \frac{\sum_{i=1}^{m}\sum_{j=1}^{n}{p}_{ij}}{n \times m\times 10}.$$

By allowing for the acceptance of some worse solutions, this acceptance criterion enables the algorithm to escape local minima and potentially find better solutions.

### Parameter optimization for ES algorithm

The proposed ES algorithm has several parameters: (a) population size, (b) Number of iterations, (c) reproduction operator, (d) selection operator, (e) mutation type, and (f) mutation rate. Population size is fixed as it is based on the reproduction operator. A large population size will provide good results but each iteration will take more computational time. Mutation operators have different types, but in this paper insertion mutation is used. The mutation rate is fixed in this paper and it is 40%. The selection operator is (µ + λ) and is fixed, so a parent can survive for many iterations unless superseded by a better offspring. So in total two parameters have to be tuned for the ES algorithm. The Multi factor analysis of variance Design of Experiments^86^ is applied for the calibration of algorithm parameters. The stopping criteria for the algorithm is 50nm milliseconds. The algorithm is tested on reeves twenty-one (21) instances i.e. Rec01, Rec03, Rec05, Rec07, Rec09, Rec11, Rec13, Rec15, Rec17, Rec19, Rec21, Rec23, Rec25, Rec27, Rec29, Rec31, Rec33, Rec35, Rec37, Rec39, and Rec41. For each instance five iterations are performed for each parameter configuration.

In the calibration phase, the computational experiment is performed on the following factors: (I) reproduction operator λ at five levels: 4, 5, 8, and 9. (II) Selection operator is at two levels: (µ + λ), (µ, λ), resulting in 4 × 2 × 21 = 168 RPD values. Table 2 shows the F-value, P-value, sum of square and mean square values for calibration phase of the ES algorithm.

**Table 2:** ANOVA results for the calibration phase of ES algorithm.

Source	Sum of squares	Df	Mean square	F-ratio	P-value
Main effects					
A: reproduction	0.005677	3	0.001892	9.72	0.0
B: selection	0.040258	1	0.040258	206.69	0.0
C: instance	0.000195	20	0.031748		

**Figure 7:**
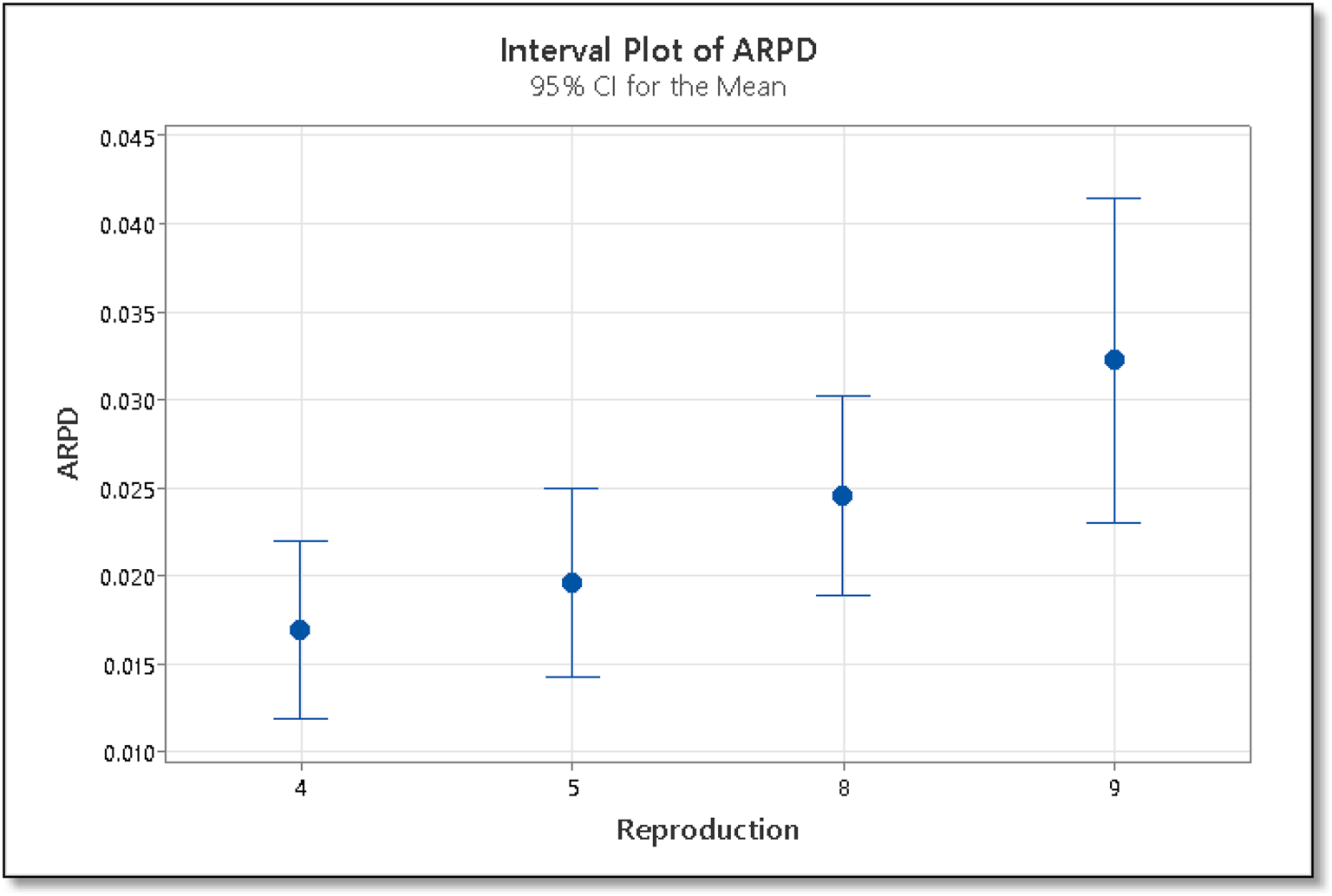
Interval plot of ARPD for various settings of reproduction operator in ES algorithm.

**Figure 8:**
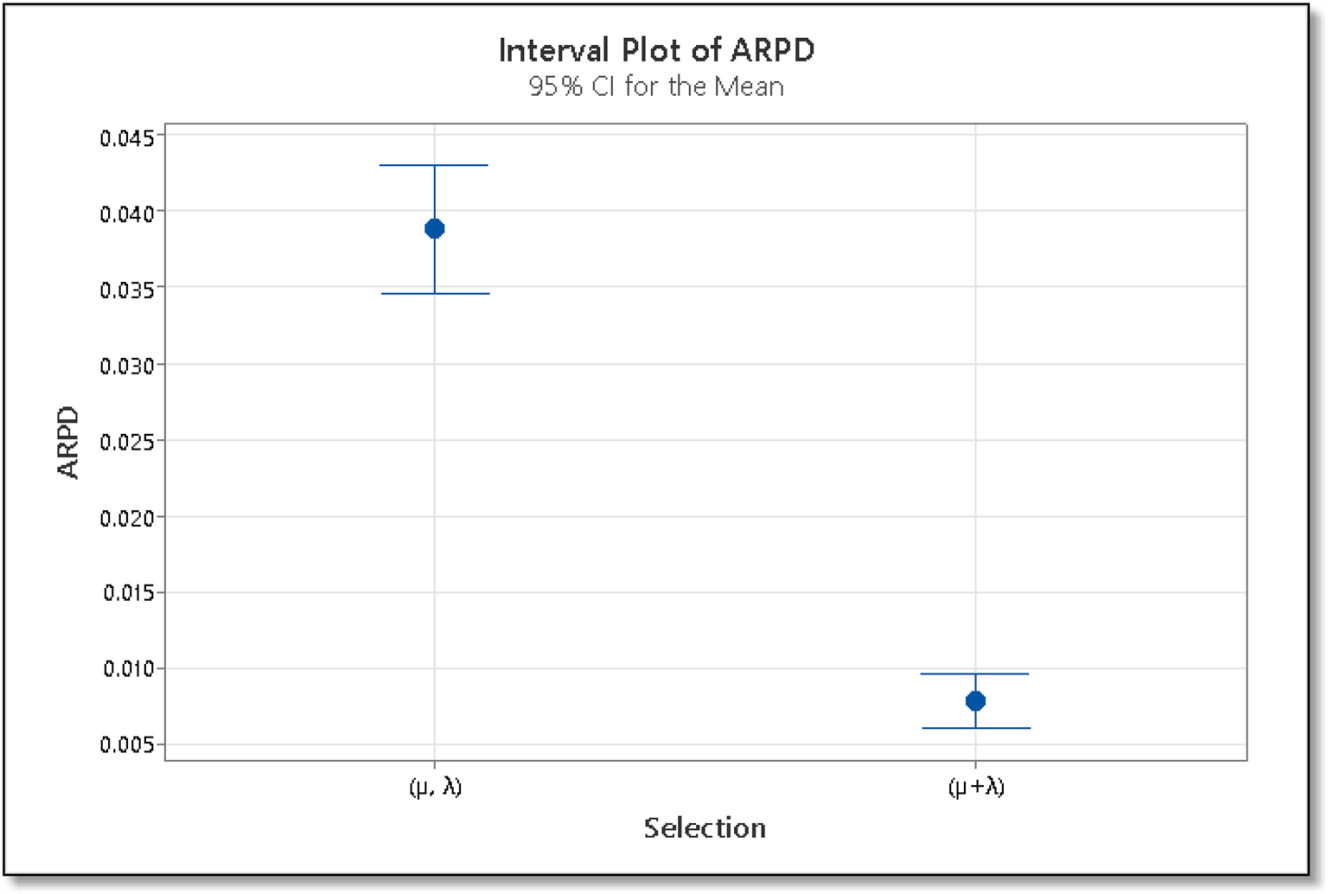
Interval plot of ARPD for various settings of selection operator in ES algorithm.

Using the ARPD as the response variable, we carry out a multi-factor ANOVA, and its results are shown in Table 3. The ARPD is calculated using Eq. (7).

7 $$ARPD=\frac{{C}^{*}-C}{C},$$

where C* is the makespan found by the ES algorithm on any instance and C is the best makespan value for that instance available in the literature. The F-value for the reproduction operator is 9.72, which shows that there is more variation between the sample means. Also, the P-value for the reproduction operator is less than 0.05, which shows that the means are significantly different. Similarly, the F-value for the selection operator is 206.69, which also proves that there is more variation between the two groups i.e. (µ + λ) and (µ, λ). The P-value for both these groups is also less than 0.05, which shows that the means are significantly different from each other. The means with 95% least significant difference confidence intervals for reproduction and mutation rate are shown in Figs. 7 and 8 respectively. If the ARPD intervals for the two means do not overlap then the difference between the two means is statistically significant. From Fig. 7, we can see that the reproduction operator value 4 is statistically better than 5, and similarly, 4 is better than 8 and 9. The reproduction value of 4 is the best among all the reproduction operators. In the case of the selection operator, From Fig. 8 we can see that the value (µ + λ) is better than (µ, λ) statistically. Hence for the ES algorithm, we will use the reproduction operator, µ = 4 and the selection operator, (µ + λ).

**Table 3:** ANOVA results for the calibration phase in IG algorithm.

Source	Sum of squares	Df	Mean square	F-ratio	P-value
Main effects					
A: Destruction	1067.14	3	355.71	42.98	0.0
B: Local search	233.70	3	77.90	9.41	0.0
C: Instance	2060.89	20	8.227

### Parameter optimization for IG algorithm

The IG algorithm has several parameters: (a) destruction, (b) construction, (c) local search, (d) acceptance-rejection criterion based on temperature and (e) number of iterations. The destruction operator has four levels i.e. d = n/10, n/15, n/2 and n/5. Construction operator depends on the destruction, as the d-jobs, which are removed during destruction, are re-inserted in the construction phase. Local search has four different methods i.e. Single insertion, multiple insertion, swap and interchange operators. Temperature for the acceptance–rejection criteria is constant and is calculated using Eq. (6). The Multi factor analysis of variance Design of Experiments^86^ is applied for the calibration of algorithm parameters. The stopping criteria for the algorithm is 50nm milliseconds. The algorithm is tested on Reeves twenty-one (21) instances i.e. Rec01, Rec03, Rec05, Rec07, Rec09, Rec11, Rec13, Rec15, Rec17, Rec19, Rec21, Rec23, Rec25, Rec27, Rec29, Rec31, Rec33, Rec35, Rec37, Rec39, and Rec41. For each instance, five iterations are performed for each parameter configuration. In the calibration phase, the computational experiment is performed on the following factors: (I) Destruction and (II) Local search, resulting in 4 × 4 × 21 = 336 RPD values. Table 3 shows the F-value, P-value, sum of square and mean square values for calibration phase of the IG algorithm.

**Figure 9:**
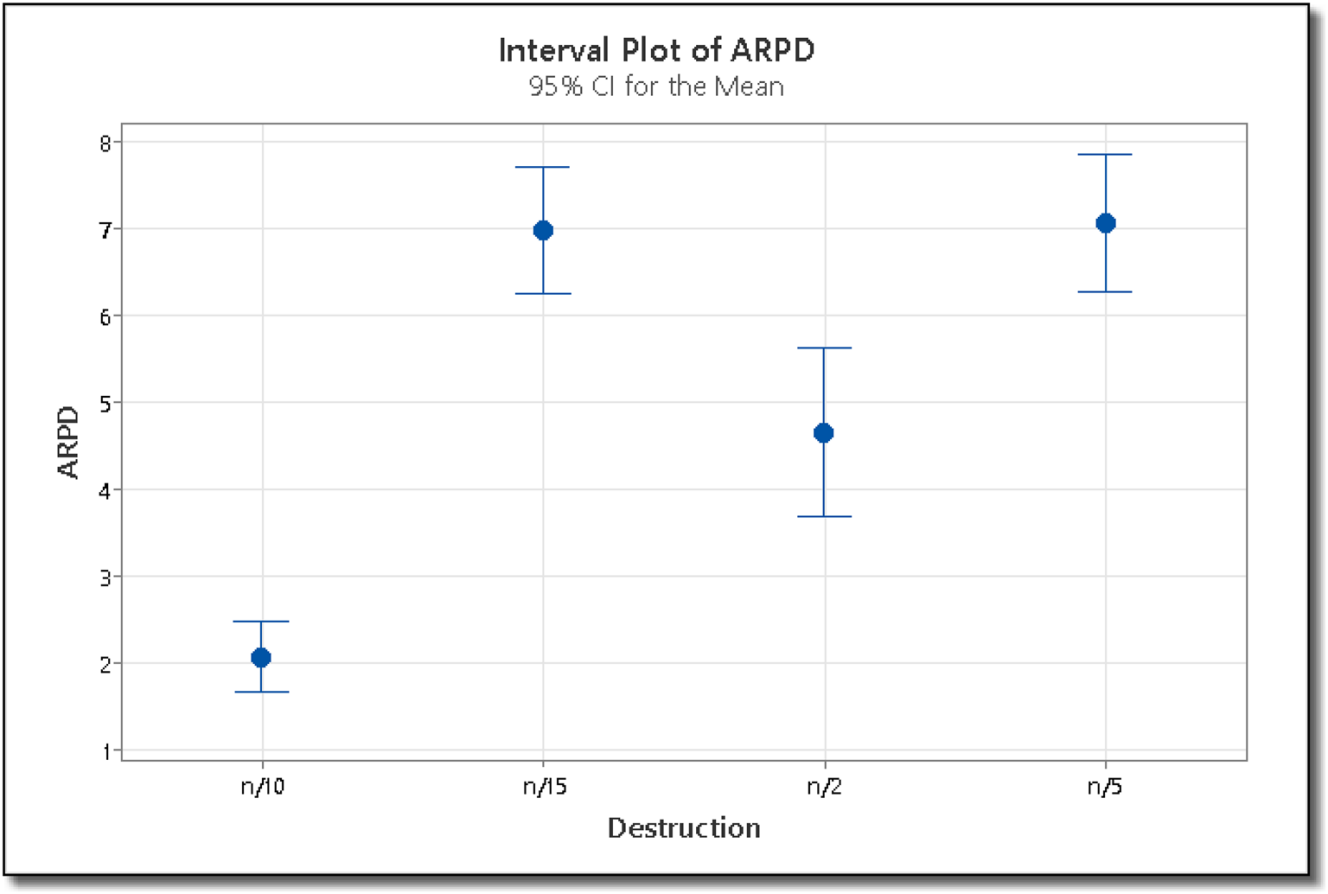
Interval plot of ARPD for various settings of destruction operator in IG algorithm.

**Figure 10:**
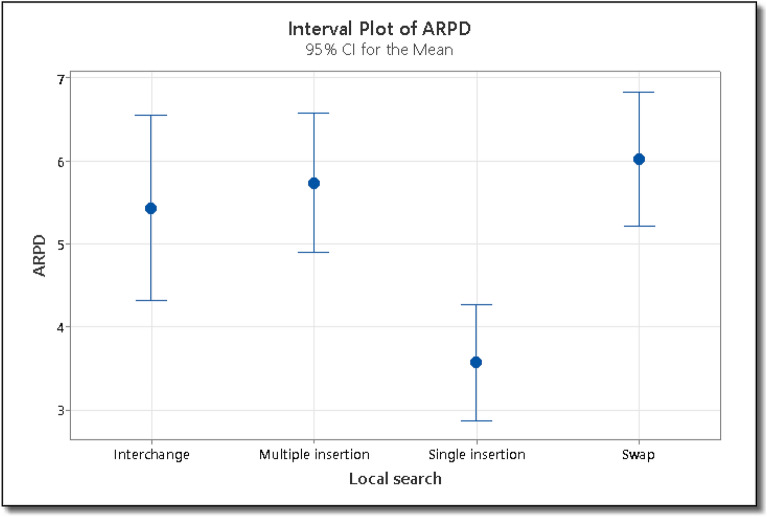
Interval plot of ARPD for various settings of local search operator in IG algorithm.

The ARPD is calculated using Eq. (7). Where C* is the makespan found by the IG algorithm on any instance and C is the best makespan value for that instance available in the literature. The F-value for the destruction and local search operator are 42.98 and 9.41 respectively. The large value of destruction operator i.e. 42.98 shows that there is more variation among sample means. The P-value for the destruction operator is less than 0.05, which shows that the means are significantly different. The F-value for the local search operator are 9.41 which shows that there is ample variation between different local search operators i.e. single insertion, multiple insertion, swap and interchange operators. The P-value for all the four groups is also then 0.05, which shows that there is significant difference between the means. The means with 95% least significant difference confidence intervals for reproduction and mutation rate are shown in Figs. 9 and 10 respectively. Since the ARPD intervals for two means do not overlap, hence there is significance difference between the two means. From Fig. 9, we can see that the for the destruction operator value n/10 is statistically better than n/2, while n/2 is better than n/15, and similarly, n/15 is better than n/5. For the destruction operator, value of n/10 is the best among all the destruction operators. In the case of the local search operator, From Fig. 10 we can see that the value single insertion is better than multiple insertion, swap and interchange operators, statistically. Hence, for the IG algorithm, we will use the destruction operator i.e. d = n/10, and the local search operator i.e. single insertion.

## Results

### Experimental setup

To validate the performance of the proposed HES-IG algorithm it is tested on Reeves (Rec)^87^ and Taillard (TA)^88^ benchmark problems. Data for the Reeves and Taillard benchmark problems is taken from the OR-library (http://people.brunel.ac.uk/~mastjjb/jeb/orlib/files/flowshop1.txt). Rec problems consist of 21 instances, where the number of jobs varies from 20, 30, 50, and 75 while the number of machines varies from 5, 10, 15, and 20. The Rec problems consist of 21 instances. The Taillard benchmark set contains 120 problems and each set is divided into 12 groups. Each group containing 10 instances, the number of machines is 5, 10, 20, and number of jobs is 20, 50, 100, 200 and 500. To validate the results of the HES-IG algorithm for NWFSSP they are compared with the algorithms of Pourhejazy et al.^34^. The author solved the NWFSSP using two heuristics i.e. Beam search (BS) and Beam search with local search (BS_LS_) algorithms. Both these algorithms were tested on Rec benchmark problems. The author showed that the BS_LS_ algorithm avoided local optimality and early convergence and found a better solution at the expense of computational time.

For a fair comparison of the HES-IG algorithm with the BS and BS_LS_ algorithms. All these algorithms were coded in MATLAB and tested on a Core™ i5 with 2.6 GHz and 4 GB memory. The computational time for each instance is set at n^2^/2 × 10 ms. Each instance was run for 30 iterations and the makespan value in Table 4 is the average value against 30 iterations.

**Table 4:** Makespan and RPD values of REC instances for BS, BS_LS_, and HES-IG algorithms.

Problem instance	Size		BS		BS_LS_		HES-IG	
	n	m	C_max_	RPD	C_max_	RPD	C_max_	RPD
rec01	20	5	1564	2.49	1555	1.90	1526	0.00
rec03	20	5	1438	5.66	1414	3.89	1361	0.00
rec05	20	5	1438	1.77	1413	0.00	1413	0.00
rec07	20	10	2062	0.98	2060	0.88	2042	0.00
rec09	20	10	2132	4.41	2108	3.23	2042	0.00
rec11	20	10	1952	3.77	1917	1.91	1881	0.00
rec13	20	15	2639	3.69	2617	2.83	2545	0.00
rec15	20	15	2573	1.74	2557	1.11	2529	0.00
rec17	20	15	2790	7.85	2644	2.20	2587	0.00
rec19	30	10	3133	9.93	2954	3.65	2850	0.00
rec21	30	10	2969	5.25	2917	3.40	2821	0.00
rec23	30	10	2921	8.19	2795	3.52	2700	0.00
rec25	30	15	3897	8.46	3724	3.65	3593	0.00
rec27	30	15	3860	12.50	3510	2.30	3431	0.00
rec29	30	15	3405	3.46	3378	2.64	3291	0.00
rec31	50	10	4642	7.78	4496	4.39	4340	0.77
rec33	50	10	4756	7.50	4543	2.69	4462	0.86
rec35	50	10	4688	6.62	4576	4.07	4422	0.57
rec37	75	20	8559	6.80	8348	4.17	8158	1.80
rec39	75	20	9033	7.28	8775	4.22	8611	2.27
rec41	75	20	9094	7.79	8853	4.93	8579	1.68

### Results comparison for Reeves benchmark problems

To compare the performance of different algorithms the relevant percentage difference (RPD) in makespan value for any instance can be calculated using Eq. (8).

8 $$\mathrm{RPD}=\frac{{\mathrm{C}}_{\mathrm{sol}}-{\mathrm{C}}_{\mathrm{best}}}{{\mathrm{C}}_{\mathrm{best}}}.$$

${C}_{best}$ is the minimum makespan value, ${C}_{sol}$ is the makespan found in ES, BS, and BS_LS_ algorithms.

A zero value of RPD shows that the algorithm has found a better upper bound while a positive value shows that the algorithm has found an inferior solution. RPD values for BS, BS_LS,_ and HES-IG algorithms are calculated using Eq. (8) and are shown in Table 4. For 20 jobs and 05 machines cases i.e. rec01, rec03, and rec05, BS_LS_ performs better than BS for all the 03 instances. However, HES-IG performs better than BS_LS_ for rec01, rec03, and rec05 where all the values of RPD for ES are zero. For 20 jobs and 10 machines cases i.e. rec07, rec09, and rec11, BS_LS_ performs better than BS for all the 03 instances, while HES-IG performs better than both BS and BS_LS_ algorithms as its RPD values are zero for all the 03 instances. For 20 jobs and 15 machines cases i.e. rec13, rec15, and rec17, BS_LS_ performs better than BS for all the 03 instances, while HES-IG performs better than both BS and BS_LS_ algorithms as its RPD values are zero for all the 03 instances.

For 30 jobs and 10 machines cases i.e. rec19, rec21, and rec23, BS_LS_ performs better than BS for all the 03 instances, while HES-IG performs better than both BS and BS_LS_ algorithms as its RPD values are zero for all the 03 instances. For 30 jobs and 15 machines cases i.e. rec25, rec27, and rec29, BS_LS_ performs better than BS for all the 03 instances, while HES-IG performs better than both BS and BS_LS_ algorithms as its RPD values are zero for all the 03 instances. Similarly, for 50 jobs and 10 machines cases i.e. rec31, rec33, and rec35, the RPD values for BS are 7.78, 7.50, and 6.62, while RPD values for BS_LS_ are 4.39, 2.69, and 4.07. Hence BS_LS_ performs better for these three instances as compared to the BS algorithm. For HES-IG, the RPD values for these instances are 0.77, 0.86, and 0.57 respectively. So HES-IG performs better than both BS_LS_ and BS algorithms.

**Figure 11:**
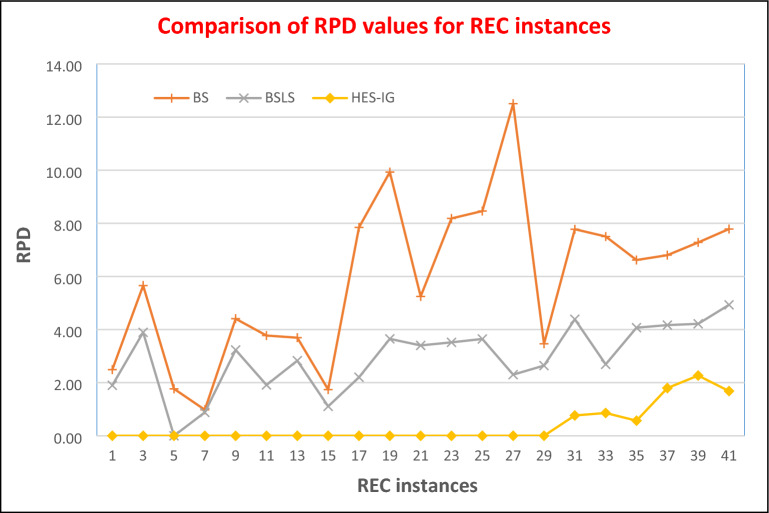
Comparison of RPD values of REC instances for HES-IG, BS and BS_LS_ algorithms.

**Figure 12:**
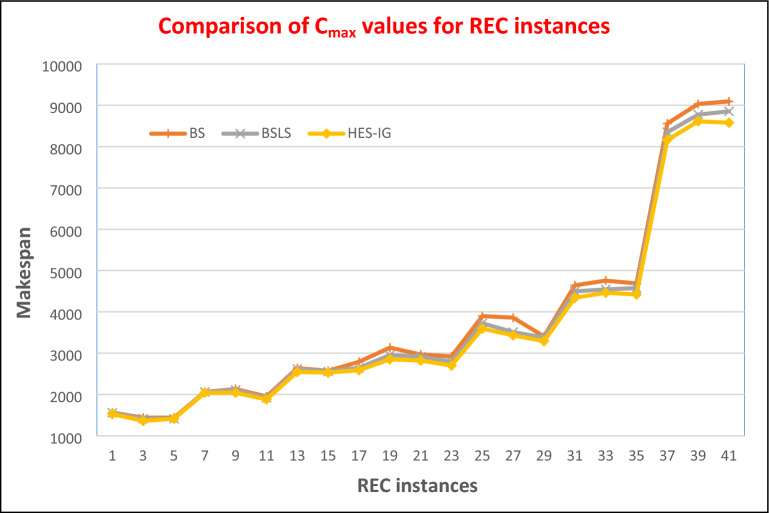
Comparison of Makespan values of REC instances for HES-IG, BS and BS_LS_ algorithms.

For the last 03 instances i.e. rec37, 39, and rec41 where we have 75 jobs and 20 machines, The RPD values for BS are 6.60, 7.28, and 7.79, while RPD values for BS_LS_ are 4.17, 4.22, and 4.93 respectively. So BS_LS_ performs better than BS for all these instances. RPD values for HES-IG for the last 03 instances are 1.80, 2.27, and 1.68 respectively. So RPD values of HES-IG are lower than the RPD values of BS and BS_LS_ algorithms, hence HES-IG performs better than BS and BS_LS_ algorithms. Therefore, the performance of HES-IG is superior as compared to BS and BS_LS_ algorithms for all the instances where the BS_LS_ algorithm performs better than the BS algorithm. The HES-IG algorithm has proven to be effective in minimizing the makespan of NWFSSP, as indicated by the results in Figs. 11 and 12. These figures show the makespan and RPD values for the BS, BS_LS_, and HES-IG algorithms across various instances of the problem, with Rec05 being an exception where BS_LS_ outperforms the other two. However, the overall trend demonstrates that the HES-IG algorithm is a robust technique that consistently outperforms the other algorithms in terms of minimizing the makespan of NWFSSP. Therefore, the HES-IG algorithm has demonstrated its effectiveness as a viable solution to the NWFSSP with makespan as the objective function.

### Results comparison for Taillard benchmark problems

To compare the performance of different algorithms on Taillard benchmark instances the relevant percentage difference (RPD) in makespan value for any instance can be calculated using Eq. (8). A zero value of RPD shows that the algorithm has found a better upper bound while a positive value shows that the algorithm has found an inferior solution. RPD values for BS, BS_LS,_ and HES-IG algorithms are calculated using Eq. (8) and are shown in Table 5. For the first group i.e. TA01–TA10, having 20 jobs and 5 machines the makespan values for HES-IG are 1486, 1528, 1460, 1588, 1449, 1481, 1483, 1482, 1469 and 1377. The makespan values for BS are 1581, 1579, 1534, 1698, 1579, 1588, 1532, 1565, 1541, and 1438, while the makespan value for BS_LS_ are 1584, 1557, 1484, 1633, 1525, 1585, 1517, 1537, 1516, and 1417 respectively. Hence, for the first group i.e. TA01-TA10, BS_LS_ algorithm performs better than BS algorithm. The RPD values of BS_LS_ are minimum as compared to BS algorithm, however none of the RPD value is zero which shows that both BS and BS_LS_ were unable to find the lower bound makespan value. Makespan values of HES-IG algorithm for TA01-TA10 are lower than both BS and BS_LS_, which shows that HES-IG algorithm performs better than both algorithms. The RPD values for HES-IG algorithm are all zero which shows that for all these instances HES-IG algorithm find the lower bound values.

**Table 5:** Makespan and RPD values of Taillard instances for BS, BS_LS_, and HES-IG algorithms.

Problem instance	Size		BS		BS_LS_		HES-IG	
	n	m	C_max_	RPD	C_max_	RPD	C_max_	RPD
TA01	20	5	1581	0.06	1548	0.04	1486	0.00
TA02	20	5	1579	0.03	1557	0.02	1528	0.00
TA03	20	5	1534	0.05	1484	0.02	1460	0.00
TA04	20	5	1698	0.07	1633	0.03	1588	0.00
TA05	20	5	1579	0.09	1525	0.05	1449	0.00
TA06	20	5	1588	0.07	1585	0.07	1481	0.00
TA07	20	5	1532	0.03	1517	0.02	1483	0.00
TA08	20	5	1565	0.06	1537	0.04	1482	0.00
TA09	20	5	1541	0.05	1516	0.03	1469	0.00
TA10	20	5	1438	0.04	1417	0.03	1377	0.00
TA11	20	10	2115	0.03	2097	0.03	2044	0.00
TA12	20	10	2270	0.05	2264	0.05	2166	0.00
TA13	20	10	1976	0.02	1975	0.02	1940	0.00
TA14	20	10	1883	0.04	1874	0.03	1811	0.00
TA15	20	10	2045	0.06	2015	0.04	1933	0.00
TA16	20	10	1999	0.06	1964	0.04	1892	0.00
TA17	20	10	2049	0.04	2012	0.02	1963	0.00
TA18	20	10	2118	0.03	2106	0.02	2057	0.00
TA19	20	10	2115	0.07	2039	0.03	1973	0.00
TA20	20	10	2159	0.05	2109	0.03	2051	0.00
TA21	20	20	3090	0.04	3077	0.03	2973	0.00
TA22	20	20	2984	0.05	2933	0.03	2852	0.00
TA23	20	20	3162	0.05	3059	0.02	3013	0.00
TA24	20	20	3206	0.07	3024	0.01	3001	0.00
TA25	20	20	3180	0.06	3151	0.05	3003	0.00
TA26	20	20	3199	0.07	3071	0.02	2998	0.00
TA27	20	20	3144	0.03	3082	0.01	3052	0.00
TA28	20	20	3054	0.08	2959	0.04	2839	0.00
TA29	20	20	3099	0.03	3099	0.03	3009	0.00
TA30	20	20	3093	0.04	3072	0.03	2979	0.00

For the second group i.e. TA11-TA20, having 20 jobs and 10 machines the makespan values for HES-IG are 2044, 2166, 1940, 1811, 1933, 1892, 1963, 2057, 1973 and 2051. The makespan values for BS are 2115, 2270, 1976, 1883, 2045, 1999, 2049, 2118, 2115 and 2159 while the makespan value for BS_LS_ are 2097, 2264, 1975, 1874, 2015, 1964, 2012, 2106, 2039 and 2109 respectively. Hence, for the second group i.e. TA11–TA20, BS_LS_ algorithm performs better than BS algorithm. The RPD values of BS_LS_ are minimum as compared to BS algorithm, however none of the RPD value is zero which shows that both BS and BS_LS_ were unable to find the lower bound makespan value. Makespan values of HES-IG algorithm for TA11–TA20 are lower than both BS and BS_LS_, which shows that HES-IG algorithm performs better than both algorithms. The RPD values for HES-IG algorithm are all zero which shows that for all these instances HES-IG algorithm find the lower bound values.

For the third group i.e. TA21-TA30, having 20 jobs and 10 machines the makespan values for HES-IG are 2973, 2852, 3013, 3001, 3003, 2998, 3052, 3009, and 2979. The makespan values for BS are 3090, 2984, 3162, 3206, 3180, 3199, 3144, 3054, 3099 and 3093 while the makespan value for BS_LS_ are 3077, 2933, 3059, 3024, 3151, 3071, 3082, 2959, 3099 and 3072 respectively. Hence, for the third group i.e. TA21–TA30, BS_LS_ algorithm performs better than BS algorithm. The RPD values of BS_LS_ are minimum as compared to BS algorithm, however none of the RPD value is zero which shows that both BS and BS_LS_ were unable to find the lower bound makespan value. Makespan values of HES-IG algorithm for TA21–TA320 are lower than both BS and BS_LS_, which shows that HES-IG algorithm performs better than both algorithms. The RPD values for HES-IG algorithm are all zero which shows that for all these instances HES-IG algorithm find the lower bound values.

**Figure 13:**
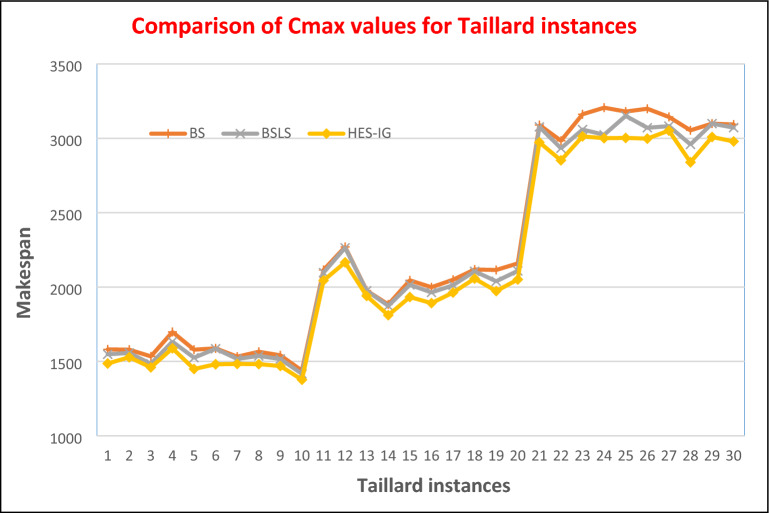
Comparison of Makespan values of Taillard instances for HES-IG algorithm with BS and BS_LS_ algorithms.

**Figure 14:**
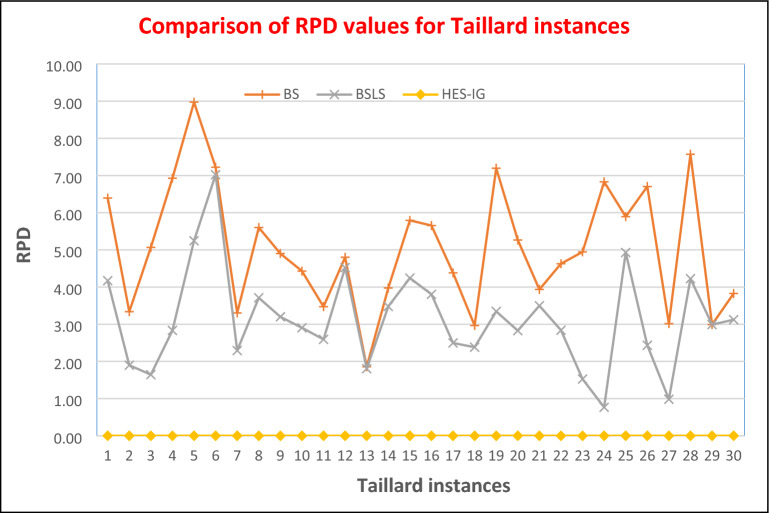
Comparison of RPD values of Taillard instances for HES-IG algorithm with BS and BS_LS_ algorithms.

From the computational comparison of all three algorithms i.e. BS, BS_LS_ and HES-IG, the performance of HES-IG is better than all other algorithms for all 30 Taillard instances. Graphical comparison against makespan values for BS, BS_LS_ and HES-IG algorithms is shown in Fig. 13, where HES-IG is performing better than the other algorithms. Graphical comparison against RPD values for BS, BS_LS_ and HES-IG algorithms is shown in Fig. 14. Hence from the testing of all algorithms on Taillard instances, HES-IG algorithm is proven to be a robust technique that consistently outperforms the other algorithms in terms of minimizing the makespan for NWFSSP.

### Wilcoxon signed rank test

Wilcoxon^89^ proposed the Wilcoxon signed-rank test for the comparison of two independent samples. To determine if two samples’ population mean ranks vary, the test compares related samples, matched samples, and performs paired difference test of repeated measurements on a single sample. Wilcoxon signed-rank test is a non-parametric statistical hypothesis test that does not assume normal distribution. Wilcoxon signed-rank test is a classical statistical method used for specific types of data. Neutrosophic statistics introduced by Smarandache^90^ is a broader framework that extends classical statistics to handle uncertainty and indeterminacy in data. It is applied in situations where there is uncertainty, vagueness, or incompleteness in the data.

In this paper, Wilcoxon signed-rank test is used to compare the performance of the HES-IG algorithm against BS and BS_LS_. This test is used to determine if there is a statistically significant difference in the performance of the algorithms. We set the significance threshold at 0.05, which means that there is a statistically significant difference between algorithms A and B if the asymptotic P-value is less than 0.05. The results of the Wilcoxon test for the HES-IG, BS, and BS_LS_ algorithms are shown in Table 6. Table 6 presents the results of the Wilcoxon signed test for comparing the HES-IG algorithm against BS and BS_LS_. It lists the Null Hypothesis and Alternative Hypothesis values for the comparison. The results indicate that the C_max_ and PI values of HES-IG are significantly different from those of BS because the P-value for HES-IG vs BS is less than 0.05. Similarly, the P-value for HES-IG vs BS_LS_ is less than 0.05, which shows that the C_max_ and PI values of HES-IG are significantly different from BS_LS_ as well. In contrast, the P-value for BS and BS_LS_ is more than 0.05, indicating that there is no statistically significant difference in C_max_ and PI values for BS and BS_LS_.

**Table 6:** Result of Wilcoxon signed test for HES-IG, BS, and BS_LS_ algorithms.

Algorithm pair	Metric	N	Median	Wilcoxon statistic	P-value	Test	
						Null hypothesis (H_o_)	Alternative hypothesis (H_1_)
HES-IG vs BS	C_max_	21	− 196	5	0.000	η = 0	η ≠ 0
	PI	41	− 1.165	327	0.182	η = 0	η ≠ 0
HES-IG vs BS_LS_	C_max_	21	− 87	14	0.000	η = 0	η ≠ 0
	PI	21	− 2.655	21	0.001	η = 0	η ≠ 0
BS vs BS_LS_	C_max_	21	110	231	0.000	η = 0	η ≠ 0
	PI	21	2.7	231	0.000	η = 0	η ≠ 0

## Conclusion, future work, and limitations

In this paper, a novel approach called the Hybrid (HES-IG) algorithm is proposed to minimize the makespan of the NWFSSP by combining the global search capabilities of the ES algorithm with the simple search abilities of the IG algorithm. The ES algorithm is a well-known numerical optimization technique that simulates natural evolution and has been successfully applied to solve a wide range of optimization problems over the last few decades. In the ES algorithm, the initial solution is generated randomly, and the mutation and reproduction operators, which are the primary sources of genetic variation, significantly impact the algorithm’s performance. To address the FSSP problem, this paper utilizes insertion mutation as it is known to be the most effective mutation operator. The reproduction operator used is (1 + 5)-ES, which generates five offspring randomly from one parent. The selection operator used is (µ + λ)-ES, which allows a good parent solution to survive for multiple generations until it is replaced by a better offspring. The results obtained from the ES algorithm are significantly enhanced through the application of the IG algorithm. To improve the quality of solutions, the IG algorithm integrates multiple operators, such as destruction, construction, local search, and acceptance–rejection criteria. The destruction operator randomly eliminates a certain number of d-jobs, which are subsequently reinserted into the partial schedule using a greedy approach to minimize the makespan. The local search operator adopts a single insertion method, and the acceptance-rejection criteria employ a constant temperature. By applying the Hybrid HES-IG algorithm to the Reeves and Taillard benchmark problems, superior computational results were achieved compared to other methods.

To enhance the performance of the HES-IG algorithm, it is suggested to use a heuristic approach to generate an initial solution for the ES algorithm. Furthermore, integrating local search methods like Simulated annealing, Tabu search, and Ant colony optimization can help to avoid local optima and speed up convergence. The efficacy of the HES-IG algorithm can be demonstrated by applying it to a practical case in an industry setting. The algorithm’s potential for success with other objective functions is also worth exploring. Additionally, it would be beneficial to test the HES-IG algorithm on challenging benchmark problems, such as Taillard and Vallada, for the NWFSSP environment. Finally, application of the HES-IG algorithm to other FSSP environments i.e. Blocking FSSP, Hybrid FSSP, Robust PFSSP, PFSSP, No-idle FSSP, etc. is expected to provide useful results.

Hybridization of ES with the IG algorithm increases the complexity and computational time of the algorithm. The initial solution in the ES algorithm is generated randomly, this reduces the computational time, but, it increases the randomness in the ES algorithm. Incorporating a Heuristic to generate the initial solution will help the ES algorithm to start with a feasible solution, and then the Hybrid algorithm can improve the solution at the expense of computational time. Wilcoxon signed-rank test is used to compare the performance of the HES-IG algorithm against BS and BS_LS_. However, if future Neutrosophic statistics can be used for validating the performances of different algorithms.
